# Pharmacological Mechanisms Involved in Sensory Gating Disruption Induced by (±)-3,4-Methylene- Dioxymethamphetamine (MDMA): Relevance to Schizophrenia

**DOI:** 10.3390/brainsci10010044

**Published:** 2020-01-13

**Authors:** Jaime Lee, Shane Thwaites, Andrea Gogos, Maarten van den Buuse

**Affiliations:** 1 Florey Institute of Neuroscience and Mental Health, University of Melbourne, Melbourne 3010, Australia; 2 School of Psychology and Public Health, La Trobe University, Melbourne 3086, Australia

**Keywords:** sensory gating, MDMA, ecstasy, dopamine, serotonin, schizophrenia

## Abstract

Sensory gating deficits have been demonstrated in schizophrenia, but the mechanisms involved remain unclear. In the present study, we used disruption of paired-pulse gating of evoked potentials in rats by the administration of (±)-3,4-methylene-dioxymethamphetamine (MDMA) to study serotonergic and dopaminergic mechanisms involved in auditory sensory gating deficits. Male Sprague-Dawley rats were instrumented with cortical surface electrodes to record evoked potential changes in response to pairs of 85dB tones (S1 and S2), 500msec apart. Administration of MDMA eliminated the normal reduction in the amplitude of S2 compared to S1, representing disruption of auditory sensory gating. Pretreatment of the animals with the dopamine D1 receptor antagonist, SCH23390, the dopamine D2 receptor antagonist, haloperidol, the serotonin (5-HT)1A receptor antagonist, WAY100635, or the 5-HT2A receptor antagonist, ketanserin, all blocked the effect of MDMA, although the drugs differentially affected the individual S1 and S2 amplitudes. These data show involvement of both dopaminergic and serotonergic mechanisms in disruption of auditory sensory gating by MDMA. These and previous results suggest that MDMA targets serotonergic pathways, involving both 5-HT1A and 5-HT2A receptors, leading to dopaminergic activation, involving both D1 and D2 receptors, and ultimately sensory gating deficits. It is speculated that similar interactive mechanisms are affected in schizophrenia.

## 1. Introduction

Patients with schizophrenia have deficits in sensory gating, which is a form of information processing [1,2]. Sensory gating refers to the ability to filter unnecessary sensory input, so that only salient information is attended to [3]. However, in schizophrenia, individuals appear to have heightened sensitivity and overflow of information input, potentially resulting in or contributing to symptoms including hallucinations and a range of cognitive deficits [3,4]. Sensory gating can be measured using various paradigms, such as P50 suppression [5,6] and the paired-click paradigm [7]. Event-related potentials (ERPs) elicited in response to sensory stimuli can be measured using electroencephalography (EEG). When two identical clicks are presented 500 milliseconds apart, the EEG response to the second stimulus (S2) is reduced when compared to the first response (S1) [8]. A reduced electrophysiological or motor response to the second click represents inhibitory gating of this relatively familiar information to allow attention to be directed towards novel, unfamiliar information; a selection mechanism which allows for an organism to function efficiently [9,10,11,12]. Several studies have found impairments in P50 suppression in schizophrenia patients and the P50 deficit is argued to be an suppression endophenotype marker of the illness [13]. Moreover, the magnitude of P50 gating disruption was associated with specific illness characteristics such as difficulties in attention, poorer working memory, and reduced processing speed [14].

The mechanisms of P50 suppression deficits seen in schizophrenia patients remain unclear. However, previous studies have suggested the involvement of both dopamine and serotonin neurotransmitters. Abnormalities in dopamine D2 [15], dopamine D1 [16], 5-HT2A [17], and 5-HT1A [18] receptor functioning have all been associated with schizophrenia. In healthy cohorts, the 5-HT2A/2C receptor agonist, N,N-dimethyltryptamine, reduced P50 suppression [19]. Moreover, schizophrenia patients treated with atypical antipsychotic medication demonstrate less P50 gating deficits compared to those treated with typical antipsychotics [20]. Combined dopamine and serotonin depletion by consumption of an amino-acid mixture deficient in L-tryptophan, L-tyrosine, and L-phenylalanine amino acids yielded a P50 gating disruption that was not present when the two neurotransmitters were depleted separately [21]. These observations suggest potential interactional mechanisms between serotonin and dopamine systems in P50 sensory gating. 

Using the same paired-click paradigm that elicits P50 suppression in humans, sensory gating is seen in rats [8,22,23]. The dopamine releaser, amphetamine, caused a disruption of auditory sensory gating in rats, most likely through activation of the dopamine D2 receptor [9] and potentially the D1 receptor [24]. Administration of the 5-HT2A receptor agonist, 1-(2,5 dimethoxy-4-iodophenyl)-2-aminopropane (DOI), or the 5-HT1A receptor agonist, 8-hydroxy-2-(di-n-propylamino) tetralin (8-OH-DPAT), have also been found to cause disruption of auditory sensory gating in rats, suggesting a role for 5-HT2A and 5-HT1A receptors in sensory gating [8,25]. Less is known on the possible interaction of serotonergic and dopaminergic involvement in this mechanism, although one study noted that the 5-HT1A receptor antagonist, UH-301, was able to block the disruption caused by amphetamine [26]. 

We previously observed that administration of the serotonin releaser, (±)-3,4 methylene-dioxymethamphetamine hydrochloride (MDMA), disrupts sensory gating in rats [25]. This disruption by MDMA was found to be greater than that elicited by DOI or 8-OH-DPAT administration, suggesting that MDMA may be acting upon both 5-HT2A and 5-HT1A receptor subtypes to cause a more robust disruption [25]. MDMA is a potent serotonin releaser, but also affects other neurotransmitter systems, such as dopamine [27]. After MDMA administration in rats, the release of dopamine was blocked by fluoxetine, a serotonin reuptake inhibitor, suggesting that serotonin may indirectly underlie the effects on the dopamine neurotransmitter system caused by MDMA [28]. The current study aimed to further investigate the mechanisms responsible for the auditory sensory gating disruption caused by MDMA. Specifically, we examined the effect of administering a pre-treatment with the dopamine D2 receptor antagonist, haloperidol, dopamine D1 receptor antagonist, SCH23390, 5-HT2A receptor antagonist, ketanserin, and 5-HT1A receptor antagonist, WAY100635, before MDMA treatment. The ultimate aim of these experiments was to obtain a better understanding of the neurotransmitter mechanisms involved in auditory sensory gating disruption deficits in schizophrenia patients.

## 2. Materials and Methods

### 2.1. Animals

Sixty male Sprague-Dawley rats were obtained from the Animal Resource Centre in Perth, WA, Australia, at approximately 200 grams. Treatments, surgical techniques, and experimental protocols were approved by the Howard Florey Institute Animal Experimentation Ethics Committee in accordance with the Australian Code of Practice for the Care and Use of Animals for Scientific Purposes (1990) established by the National Health and Medical Research Council of Australia. 

Rats were kept in the animal house facility at the Florey Institute of Neuroscience and Mental Health (Parkville, Victoria, Australia). The rats were initially housed in pairs in standard rat cages with ad libitum access to food pellets and water within a holding room that was maintained at 19–22 °C. A 12 h light/dark cycle was kept, where the lights were on between 7 am and 7 pm. Post-surgery, the rats were housed individually to avoid any complications. 

### 2.2. Surgery

The electrode implantation procedure was based on methodology described previously [8,29]. Briefly, this involved stereotaxic surgery to implant electrodes onto the dura mater of the rat cortex, when the rats were between 250 and 350 grams. Rats were anaesthetised with 5% isoflurane and mounted into a stereotaxic frame with a heating pad maintained at 37 °C. A non-steroidal anti-inflammatory, Carprofen (Rimadyl®, Pfizer Ltd., Sandwich, Kent, UK), was injected at 5 mg/kg. A small area of the head was then shaved and approximately 0.1 mL of Lignocaine (Pfizer, NSW, Australia) was injected under the skin on the head as a local anaesthetic. A 2 cm incision was then made on the head and the bregma, was located. For the recording electrode, one hole was drilled 4 mm posterior and 1 mm lateral to the midline. Holes for the reference and earth electrodes were 1 mm anterior and ± 1 mm lateral from bregma, respectively. Three additional holes around the electrode holes were drilled for anchor screws (0-80 × 3/32, PlasticsOne, Roanake, VA, USA). A pedestal (MS 363, PlasticsOne) and a dust cap (363DC, PlasticsOne) were also used. 

The earth and reference electrode tips were 2 mm in length and 0.125 mm in diameter (part number E363/1) and the recording electrode was 5 mm in length and 0.25 mm in diameter (part number E363/3). The headpiece, consisting of the pedestal and electrodes, was inserted and dental cement (Vertex-Dental, Zeist, The Netherlands) was used to secure it onto the skull of the rat. After the cement had dried and hardened, the stereotaxic electrode holder was removed from the headpiece and the surrounding skin was sutured closed. A dust cap was secured onto the head plug to avoid any particles blocking the holes in the pedestal and to keep the area clean, and antiseptic cream (Betadine®, povidone-iodine 10% w/w, Faulding Consumer, Salisbury, SA, Australia) was applied to the surrounding skin. The rats were then transferred from the stereotaxic frame into a clean, heated recovery box at 30 °C. When the rats were conscious and mobile, they were placed individually into a clean standard rat cage. 

### 2.3. ERP Testing

#### 2.3.1. Apparatus

The recording chamber consisted of a timber box (44 cm high × 40 cm wide × 40 cm deep) that was lined with foil and connected to an earth point acting as an electromagnetic shield. The rats were placed inside a clear plexiglass cylinder in the middle of the box and plugged into an electronic swivel that allowed freedom of movement. Electromagnetically-shielded speakers were placed against an opening on both sides of the box that was covered with fine aluminium mesh to allow the white noise and clicks to be presented to the rats while maintaining electromagnetic shielding. A third hole at the top of the box, also covered with aluminium mesh, allowed light into the box. An amplifier and laptop computer were connected to the recording chamber to control the presentation of the sounds, as well as record and acquire the data. The amplifier gain was set to 10,000× and high and low pass filters were set to 0.05 Hz and 300 Hz, respectively. 

#### 2.3.2. ERP Testing Sessions

Two weeks after surgery, the rats were presented with a full session of white noise and click to acclimatise them to the recording chamber. A series of four experimental pretreatment/treatment sessions commenced 2–3 days later and 2–3 days between sessions to allow drug washout. In each animal, sessions included MDMA only, antagonist only, antagonist plus MDMA, or no drug treatment. A single testing session of 150 individual trials lasted approximately 25 min, including a three min acclimation period. The acclimation period consisted of 65 dB white noise, which was also present between trials. Two identical 85 dB clicks were presented 500 ms apart for each trial. Each trial was separated with random intervals of between 5 and 9 s to minimise the chances of a learned response or expectation of a stimulus impacting upon the results. EEG was measured 200 ms prior to the first click and until 500 ms after the second click.

#### 2.3.3. ERP Signal Processing

The EEGLAB plugin [30] for Matlab was used to import raw epoch data in order to visually inspect all 150 trials. Each epoch was filtered with a 0.1 Hz high-pass filter and the baseline was removed on the basis of the pre-stimulus data. Each epoch was individually inspected and rejected if gross movement or amplifier saturation artefacts were present. The remaining epochs were averaged together to form a single waveform. P1 and N1 components were analysed from this waveform. The P1 amplitude was measured as the highest peak between 0–80 ms after the stimulus, where N1 amplitude was the most negative trough that immediately followed P1. S2:S1 ratios were obtained by P1 minus N1 amplitudes that occurred after the second click (S2 amplitude) divided by the P1 minus N1 that occurred after the first click (S1 amplitude). S2:S1 ratios below 0.6 were considered to be indicative of normal gating mechanisms.

### 2.4. Drugs

All drugs were diluted and prepared to an injection volume of 1 mL/kg on the day of the testing session. Drug doses used were in accordance with previous literature. A 2 mg/kg dose of MDMA ((±) 3,4 methylene-dioxymethamphetamine hydrochloride, Sigma Aldrich, St. Louis, MO, USA) was injected intraperitoneally. This was previously shown to elicit a paired-pulse sensory gating deficit [25]. Pretreatment drugs, haloperidol (Serenace®, Aspen Pharma Pty Ltd, St Leonards, NSW, Australia) 0.25 mg/kg, SCH23390 (Tocris Bioscience, Bristol, UK) 0.1 mg/kg, ketanserin tartrate (Tocris Bioscience, Bristol, UK) 2 mg/kg and WAY100635 maleate (Tocris Bioscience, Bristol, UK) 1 mg/kg, were all injected subcutaneously. The doses of these drugs were chosen on the basis of literature and preliminary experiments. Saline was injected subcutaneously for pretreatment control, but intraperitoneally for the treatment control injection. The pretreatment drug (saline or antagonist) was administered 30 min prior to the treatment drug (saline or MDMA). Recordings began 10 min after the treatment injection to allow time for the drug to take its effects and for the rat to settle into the chamber. 

### 2.5. Experimental Design and Analysis

Rats were allocated into one of the four antagonist groups and the treatment sequence within each group was pseudorandomised. A 2 × 2 repeated measures design was implemented, where the rats were either injected with saline or the antagonist as the pretreatment, then saline or MDMA as the treatment injection. Data were analysed using Systat 13.0 (SPSS Science, Chicago, IL, USA). A one-way analysis of variance (ANOVA) was used to analyse the S2:S1 ratio with repeated measures applied for the antagonist treatment (saline or antagonist) and treatment (saline or MDMA). A repeated measures ANOVA was also conducted to analyse the P1-N1 amplitudes for both S1 and S2 responses, with the addition of trial type (S1 or S2) variable. Alpha was set at 0.05 for all statistical analyses. 

## 3. Results

### 3.1. Animals

All sixty rats (mean = 297grams) underwent surgery, however, seventeen rats were excluded from the final analyses due to either incomplete experiments or lack of normal baseline gating, indicated by a saline/saline S2:S1 ratio of above 0.6. In the remaining cohort of 43 rats, ten received haloperidol pretreatment, eight received SCH23390 pretreatment, 13 received ketanserin pretreatment and 12 were pretreated with WAY100635.

**Figure 1:**
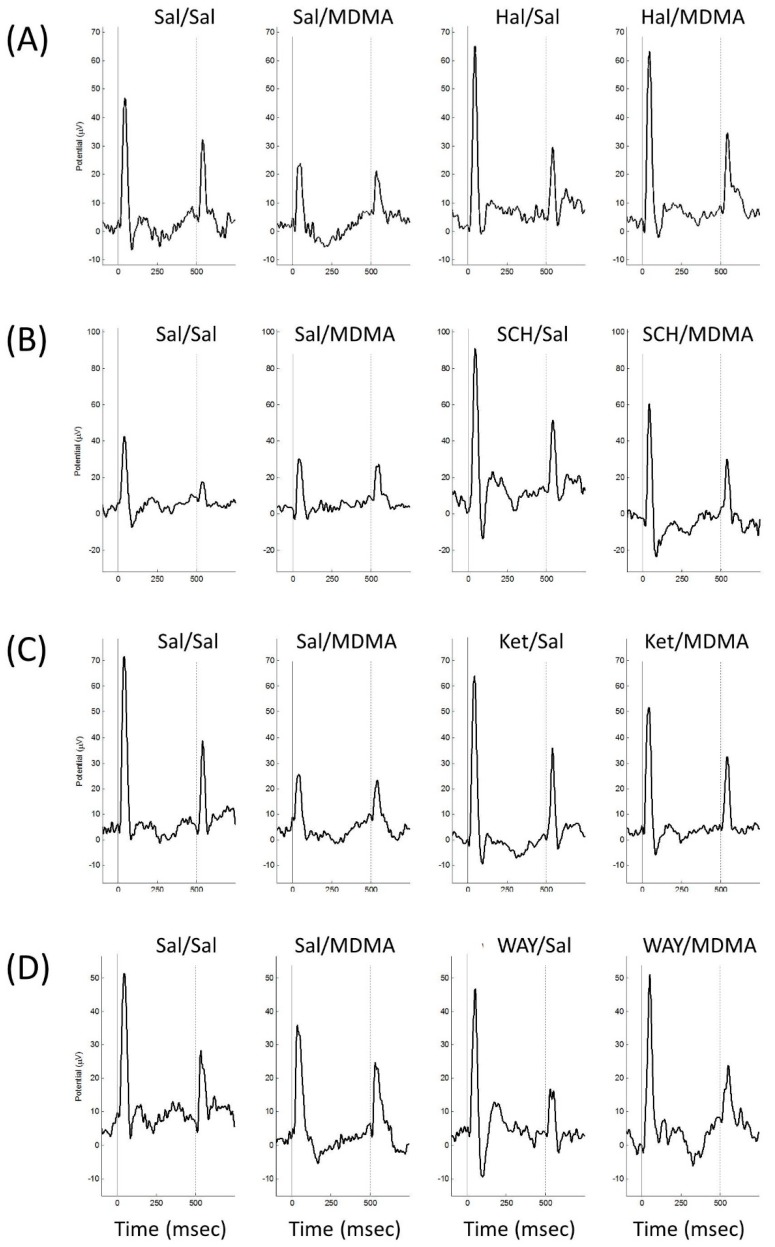
Grand-average waveforms for each MDMA cohort and condition. Clear event-related potentials (ERP) from the two clicks, indicated by the grey vertical lines, were elicited for each cohort in each condition. (**A**) shows the grand-average waveform for the haloperidol (Hal) cohort. (**B**) displays the grand-average waveforms for the SCH23390 (SCH) cohort. (**C**) shows the grand-average waveforms for the ketanserin (Ket) cohort. (**D**) displays the grand-average waveforms for the WAY100635 (WAY) cohort.

Grand-average waveforms indicate clear ERPs elicited by the presentation of the first (S1) and the second (S2) stimulus (Figure 1). A prominent positive peak was labelled as P1 (mean latency for S1 = 42.5 ms, mean latency for S2 = 41.3 ms) and the negative trough as N1 (mean latency for S1 = 83.1 ms, mean latency for S2 = 76.6 ms). 

### 3.2. MDMA

#### 3.2.1. S2:S1 Ratio

In the combined cohort of 43 rats, MDMA administration consistently resulted in a disruption of sensory gating. The S2:S1 ratio from the MDMA treatment condition (mean = 0.74) was significantly higher than the saline treatment condition (mean = 0.47) (F(1,42) = 291.3, *p* < 0.001). 

#### 3.2.2. P1-N1 Amplitude

**Figure 2:**
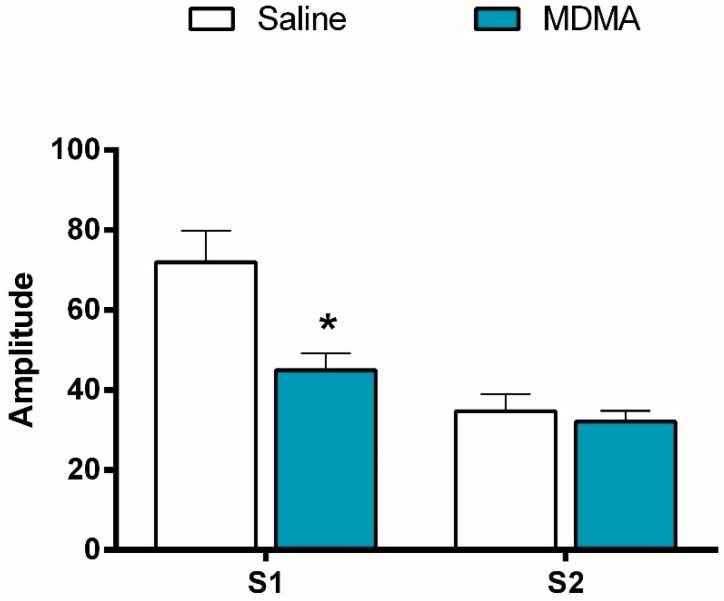
(±)-3,4-methylene-dioxymethamphetamine (MDMA) disrupted auditory sensory gating via a reduction in the S1 amplitude. Mean S1 and S2 amplitudes for all saline/saline and saline/MDMA conditions are displayed with error bars indicating standard error of the mean (SEM). * denotes significance at *p* < 0.05. *N* = 43.

The effect of MDMA on gating was due to alterations in the S1 response, rather than the S2 response (Figure 2). There was a significant MDMA and trial type interaction (F(1,42) = 56.4, *p* < 0.001). When analysing S1 alone, the amplitude was significantly lower with MDMA treatment compared to saline treatment (F(1,42) = 16.8, *p* < 0.001, Figure 2). However, S2 responses remained unchanged with the two treatments (Figure 2). 

### 3.3. Effect of Haloperidol Pretreatment on MDMA-Induced Sensory Gating Disruption

#### 3.3.1. S2:S1 Ratio

**Figure 3:**
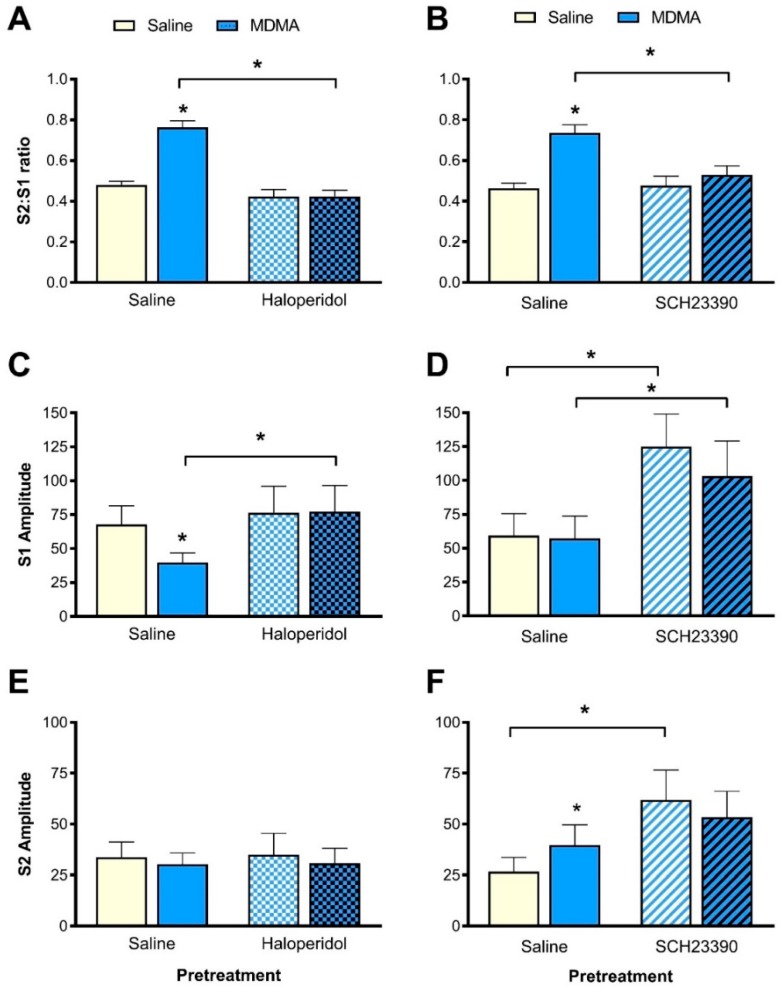
Effect of haloperidol (**A**,**C**,**E**) or SCH23390 (**B**,**D**,**F**) on MDMA-induced disruption of auditory sensory gating. (**A**,**B**) MDMA increased the S2:S1 ratio, an effect that was blocked by haloperidol and SCH23390 pretreatment, respectively. (**C**) MDMA treatment reduced the S1 amplitude and pretreatment with haloperidol prevented this effect. (**D**) SCH23390 pretreatment increased S1 amplitude for both saline and MDMA treatments. (**E**) S2 amplitudes remained unaffected by MDMA and haloperidol. (**F**) MDMA slightly increased S2 amplitude in the saline pretreatment condition. SCH23390 pretreatment prevented this effect and increased S2 amplitude. Data are mean ± standard error of the mean (SEM). * denotes significance at *p* < 0.05. *N* = 10 and 8, respectively.

MDMA increased the S2:S1 ratio and this effect was blocked by haloperidol pretreatment (Figure 3A). ANOVA revealed a significant interaction between haloperidol pretreatment and MDMA treatment for the S2:S1 ratio (F(1,9) = 21.4, *p* = 0.001, Figure 3A). Further analysis indicated that when the rats were pretreated with saline, MDMA treatment significantly increased the S2:S1 ratio (F(1,9) = 96.7, *p* < 0.001), indicating disruption of sensory gating. However, after pretreatment with haloperidol, there were no differences in the S2:S1 ratio between saline- or MDMA-treatment conditions. Moreover, further pairwise analysis indicated that the S2:S1 ratio was significantly lower in the MDMA treatment condition after haloperidol pretreatment compared to saline pretreatment (F(1,9) = 34.6, *p* < 0.001, Figure 3A).

#### 3.3.2. P1-N1 Amplitude

The interaction of haloperidol pretreatment with the effect of MDMA was more prominent on the S1 compared to the S2 amplitude (Figure 3C,E). There were significant interactions of pretreatment, MDMA dose and trial type (F(1,9) = 17.2, *p* = 0.003), as well as haloperidol and trial type (F(1,9) = 9.6, *p* = 0.01) and MDMA and trial type (F(1,9) = 8.2, *p* = 0.02), while there was a trend towards significance for a haloperidol and MDMA interaction (F(1,9) = 5.1, *p* = 0.051). 

When analysing S1 alone (Figure 3C), there was a significant interaction for haloperidol pretreatment and MDMA treatment (F(1,9) = 13.2, *p* = 0.005). When rats were pretreated with saline, MDMA treatment caused a significant reduction in S1 amplitude compared to saline treatment (F(1,9) = 11.6, *p* = 0.008) but there were no differences in S1 amplitude for the haloperidol- pretreatment conditions. In addition, while haloperidol did not alter S1 amplitude on its own, the S1 amplitude in the MDMA-treatment condition was higher after haloperidol pretreatment compared to saline pretreatment (F(1,9) = 8.8, *p* = 0.02, Figure 3C), consistent with haloperidol blocking the action of MDMA on S1. The S2 amplitude was not affected by haloperidol pretreatment or MDMA treatment (Figure 3E). 

### 3.4. Effect of SCH23390 Pretreatment on MDMA-Induced Sensory Gating Disruption

#### 3.4.1. S2:S1 Ratio

**Figure 4:**
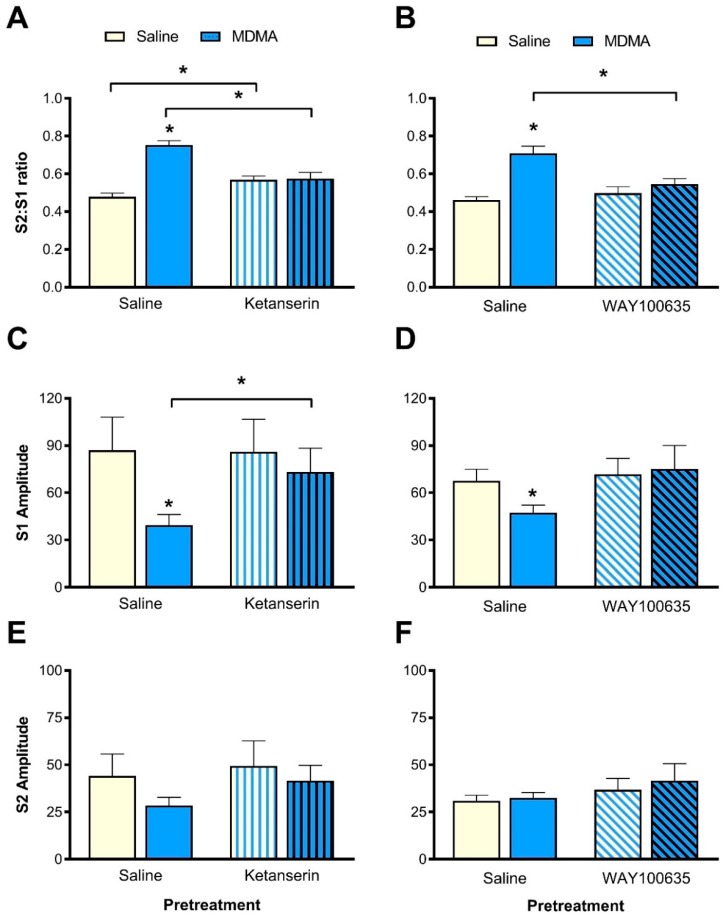
Effect of ketanserin (**A**,**C**,**E**) or WAY100635 (**B**,**D**,**F**) on MDMA-induced disruption of auditory sensory gating. (**A**,**B**) MDMA increased the S2:S1 ratio, an effect which was blocked by both ketanserin and WAY100635 pretreatment, respectively. (**C**,**D**) MDMA treatment reduced the S1 amplitude and this effect was blocked by pretreatment with ketanserin or WAY100635, respectively. (**E**,**F**) S2 amplitudes remained unaffected by MDMA, ketanserin or WAY100635. Data are mean ± standard error of the mean (SEM). * denotes significance at *p* < 0.05. *N* = 13 and 12, respectively.

S2:S1 ratio was increased with MDMA treatment and SCH23390 pretreatment was able to block this effect (Figure 3B). ANOVA revealed a significant interaction between SCH23390 pretreatment and MDMA treatment for the S2:S1 ratio (F(1,7) = 18.9, *p* = 0.003, Figure 4A). When rats were pretreated with saline, MDMA treatment significantly increased the S2:S1 ratio (F(1,7) = 53.9, *p* < 0.001), indicative of a disruption in sensory gating. However, with SCH23390 pretreatment, saline and MDMA treatment did not alter the S2:S1 ratio. Furthermore, when comparing the MDMA-treatment conditions, pretreatment with SCH23390 resulted in a significantly lower S2:S1 ratio compared to saline pretreatment (F(1,7) = 25.4, *p* = 0.001, Figure 3B).

#### 3.4.2. P1-N1 Amplitude

The interaction between SCH23390, MDMA and trial type was not significant. However, the interactions between SCH23390 and trial type (F(1,7) = 29.5, *p* = 0.001) and MDMA and trial type (F(1,7) = 7.8, *p* = 0.03) were significant, while there was a trend towards significance for the SCH23390 and MDMA interaction (F(1,7) = 5.3, *p* = 0.055) (Figure 3D,F).

When analysing S1 alone (Figure 3D), there was no significant interaction between SCH23390 and MDMA. In this cohort of rats, MDMA or saline treatment did not alter the S1 responses. However, there was a significant main effect of SCH23390 pretreatment (F(1,7) = 23.1, *p* = 0.002). Further pairwise comparisons revealed a significantly higher S1 amplitude with SCH23390 pretreatment compared to saline pretreatment for both the saline-treatment (F(1,7) = 24.5, *p* = 0.002) and MDMA-treatment (F(1,7) = 12.4, *p* = 0.01, Figure 3D) conditions. 

In these animals, in contrast to the S1 amplitudes, ANOVA of S2 amplitudes revealed a significant interaction between SCH23390 and MDMA (F(1,7) = 9.5, *p* = 0.02, Figure 3F). Further analysis indicated that when rats were pretreated with saline, MDMA treatment slightly, but significantly increased the S2 response (F(1,7) = 7.5, *p* = 0.03), however, in the SCH23390- pretreatment conditions, the S2 response remained unchanged. Similarly, the S2 responses in the MDMA-treatment conditions were not altered by the pretreatment. On the other hand, the S2 responses in the saline-treatment conditions were significantly higher with SCH23390 pretreatment compared to saline pretreatment (F(1,7) = 10.0, *p* = 0.02, Figure 3F). 

### 3.5. Effect of Ketanserin Pretreatment on MDMA-Induced Sensory Gating Disruption

#### 3.5.1. S2:S1 Ratio

MDMA caused an increase to the S2:S1 ratio, which was blocked by ketanserin (Figure 4A). There was a significant interaction between ketanserin pretreatment and MDMA treatment for the S2:S1 ratio (F(1,12) = 45.1, *p* < 0.001, Figure 4A). When rats were pretreated with saline, MDMA treatment significantly increased the S2:S1 ratio compared to saline treatment (F(1,12) = 71.2, *p* < 0.001), indicating that sensory gating was disrupted. In contrast, MDMA or saline treatment did not affect the S2:S1 ratio after ketanserin pretreatment. With MDMA treatment, ketanserin pretreatment resulted in significantly lower S2:S1 ratio compared to saline pretreatment (F(1,12) = 27.9, *p* < 0.001). In addition, further pairwise analyses revealed that ketanserin pretreatment slightly, but significantly increased the S2:S1 ratio when rats were treated with saline (F(1,12) = 10.4, *p* = 0.01, Figure 4A). 

#### 3.5.2. P1-N1 Amplitudes

The interaction between ketanserin pretreatment and MDMA treatment was due to changes to S1 amplitude rather than changes to S2 amplitude (Figure 4C,E). There was a significant interaction between ketanserin pretreatment, MDMA treatment and trial type (F(1,12) = 6.9, *p* = 0.02), as well as a significant interaction between MDMA treatment and trial type (F(1,12) = 14.4, *p* = 0.003). 

When analysing S1 amplitudes alone (Figure 4C), the interaction between ketanserin pretreatment and MDMA treatment was not significant. However, there was a significant main effect of MDMA treatment (F(1,12) = 6.1, *p* = 0.03). MDMA treatment resulted in a significant reduction of S1 amplitude compared to saline treatment (F(1,12) = 6.9, *p* = 0.02, Figure 4C). However, there were no changes to the S1 amplitude in the ketanserin-pretreatment conditions. Similarly, the S1 amplitude remained unaltered with saline and ketanserin-pretreatment in the saline-treatment condition. In contrast, in the MDMA-treatment conditions, ketanserin pretreatment resulted in significantly greater S1 amplitudes compared to saline pretreatment (F(1,12) = 11.8, *p* = 0.005, Figure 4B). In contrast to S1, there were no significant effects of ketanserin pretreatment on the S2 amplitude (Figure 4E). 

### 3.6. Effect of WAY100635 Pretreatment on MDMA-Induced Sensory Gating Disruption

#### 3.6.1. S2:S1 Ratio

MDMA treatment resulted in an increase in the S2:S1 ratio, which was blocked with WAY100635 pretreatment (Figure 4B). ANOVA revealed a significant interaction between WAY100635 pretreatment and MDMA treatment (F(1,11) = 11.6, *p* = 0.006, Figure 4B). When rats were treated with saline, MDMA treatment significantly increased the S2:S1 ratio compared to saline treatment (F(1,11) = 66.0, *p* < 0.001), demonstrating a sensory gating disruption. However, when rats were pretreated with WAY100635, there was no difference in the S2:S1 ratio between saline and MDMA treatment. In addition, in the MDMA-treatment condition, there was a significantly lower S2:S1 ratio when rats were pretreated with WAY100635 compared to saline pretreatment (F(1,11) = 15.7, *p* = 0.002, Figure 4B). 

#### 3.6.2. P1-N1 Amplitude

The effect of MDMA-induced sensory gating disruption was due to changes to the S1, rather than the S2 (Figure 4D,F). There was a significant interaction between WAY100635 pretreatment, MDMA treatment and trial type (F(1,11) = 9.0, *p* = 0.012), as well as a significant MDMA x trial type interaction (F(1,11) = 25.8, *p* < 0.001). 

When analysing S1 and S2 alone, there were no significant interactions between WAY100635 pretreatment and MDMA treatment. However, pairwise comparison between saline-pretreatment conditions revealed that the S1 amplitude was significantly lower for MDMA treatment compared to saline treatment (F(1,11) = 8.1, *p* = 0.016, Figure 4D), whereas the S2 amplitude remained unchanged between these two conditions (Figure 4F). Further analysis revealed no significant differences between MDMA-treatment or saline-treatment conditions for both S1 and S2 amplitudes after WAY100635 pretreatment (Figure 4D,F).

## 4. Discussion

This study examined the role of dopamine and serotonin receptors in the disruption of paired-pulse sensory gating caused by MDMA in rats. Antagonist drugs acting at dopamine D1, dopamine D2, 5 HT1A and 5-HT2A receptors were all effective in reversing the sensory gating deficit induced by MDMA administration. These findings suggest that dopamine D1 and D2, and 5-HT1A and 5 HT2A receptors may act in series and synergistically which results in any one of the antagonists inducing a blockade of auditory sensory gating disruption caused by MDMA. 

### 4.1. In-Series Involvement of Serotonin and Dopamine Pathways 

The fact that antagonism of each of the four receptors that were investigated in the current study completely blocked the MDMA effect suggests that the serotonin and dopamine pathways act in series and not parallel to one another. If they were to act in parallel, only partial blockade of the MDMA disruption would be seen with each antagonist treatment. Though speculative, the current data supports literature, which suggests that the dopaminergic pathways that are involved in the regulation of sensory gating may be downstream of serotonergic neurons [28,31].

Given that MDMA is primarily a serotonin releaser, it is perhaps unsurprising that the 5-HT receptor antagonists were able to completely restore sensory gating after disruption caused by MDMA. However, the results that both of the dopamine receptor antagonists, haloperidol and SCH23390, were also able to completely block the MDMA-induced disruption to auditory sensory gating is somewhat surprising considering that the effects of MDMA on the dopamine system have been reported to be less potent than on the serotonin system. For instance, Stone et al., [32] found that within 15 minutes of acute MDMA administration, there was a decrease in the levels of the serotonin precursor enzyme, tryptophan hydroxylase, in the neostriatum, frontal cortex, hippocampus and hypothalamus of the rat, attributed to the rapid release and activity of serotonin induced by MDMA. On the other hand, MDMA did not alter the activity of tyrosine hydroxylase, but dopamine levels were temporarily increased in the neostriatum within the same time period [32]. Moreover, Biezonski et al. [31] reported that one week after a 10mg/kg binge of MDMA administered once every hour for 4 hours, serotonin concentrations in the striatum and frontal cortex were reduced. However, there was no significant reduction of dopamine levels. Lastly, Oakly et al. [33] showed that in serotonin transporter knockout rats, the reinforcing properties of MDMA were markedly enhanced, illustrating an important primary effect of MDMA on serotonergic neurons in rats [33]. Despite all of the above reports, the current results indicate that the dopaminergic system is also implicated, at least secondarily, in the mechanism of action of MDMA in addition to the serotonergic system.

Previous studies support the hypothesis that the action of ketanserin in restoring sensory gating could involve downstream effects on the dopamine system, particularly in the VTA and nucleus accumbens. Bortolozzi et al. [34] found that 5-HT2A receptors in the medial prefrontal cortex mediated dopamine release in the medial prefrontal cortex and the VTA. The authors found that administration of DOI resulted in increased activity in 65% of the dopaminergic VTA neurons recorded. Moreover, Orejarena et al. [35] found that compared to wildtype mice, 5-HT2A receptor knockout mice have reduced mesolimbic dopaminergic activity, particularly in the nucleus accumbens, which resulted in behavioural deficits in self-administration of MDMA. Interestingly, this receptor knockout did not affect d-amphetamine or cocaine self-administration, suggesting that the 5-HT2A receptor is selectively implicated in the action of this serotonin releaser, MDMA [35]. Therefore, it is possible that in the current study ketanserin reduced a 5-HT2A-mediated increase in dopaminergic transmission, which may have blocked MDMA-induced sensory gating deficits. 

5-HT2A receptors have also been found to act upstream to dopamine D1 receptors [36,37]. Scarlota et al. [36] found that DOI induced head bobs in rabbits, which were reduced with SCH23390 pretreatment. As DOI has low affinity for dopamine receptors, it is likely that the dopamine D1 receptor involvement is due to a downstream effect from the 5-HT2A receptors. Therefore, it is possible that in the current study, blockade of 5-HT2A receptor activity also indirectly blocked enhanced dopamine D1 receptor activation, which prevented MDMA from causing a disruption to sensory gating. 

Similar to 5-HT2A receptors, post-synaptic 5-HT1A receptors have been postulated to modulate dopamine transmission, particularly in the prefrontal cortex and VTA [38,39]. Ichikawa et al. [39] found that 8-OH-DPAT increased dopamine release in the prefrontal cortex of male Sprague-Dawley rats, effects that were reversed by WAY100635. Increases in dopamine activity in cortical regions were also induced with various atypical antipsychotics with partial 5-HT1A receptor agonist activity, such as ziprasidone. Striatal dopamine release has been found to be unaltered by WAY100635 or 8-OH-DPAT treatment [40]. Therefore, the mesocortical dopamine pathways and prefrontal dopamine activity may be activated by 5-HT1A receptors. In the current study, administration of WAY100635 may have decreased the cortical dopaminergic activation associated with MDMA administration through blockade of post-synaptic 5-HT1A receptors, which prevented a disruption of auditory sensory gating. It is unclear which dopamine receptor subtype is targeted by this increase in cortical dopamine release. However, it is more likely to be dopamine D1 receptors rather than dopamine D2 receptors, as they are more prevalent in the prefrontal cortex. Furthermore, Diaz-Mataix et al. [38] found that administration of the 5-HT1A agonist, BAYx3702, affected dopamine release in the VTA in addition to the medial prefrontal cortex. There is a high density of 5-HT1A receptors in the area of the medial prefrontal cortex that projects to the VTA, which also contains 5-HT2A and dopamine D2 receptors [38]. It is, thus, possible that activation of 5-HT1A receptors results in complex indirect stimulation of dopamine D1 receptors in the prefrontal cortex, as well as downstream dopamine D2 and/or 5-HT2A receptors in the VTA. Conversely, WAY100635 pretreatment may have inhibited downstream dopaminergic activity through lack of activation of the downstream dopamine D1, dopamine D2 and/or 5-HT2A receptors, and, thus, MDMA effects were blocked. 

Although the primary mechanism of action of MDMA is likely enhanced serotonin release via the serotonin transporter, clearly dopaminergic mechanisms are also involved. Haloperidol pretreatment was effective in completely blocking the MDMA-induced auditory sensory gating deficits through prevention of a S1 amplitude decrease. It has been previously shown by Adler et al. [9] that haloperidol reverses sensory gating deficits induced by amphetamine and the current study demonstrates that dopamine D2 receptors also play a crucial role in the effects of MDMA. de Bruin et al. [41] found that auditory sensory gating can be disrupted by administration of the dopamine D2 receptor agonist, quinpirole, directly into the nucleus accumbens. While the current study did not employ localised injections, it is possible that MDMA disrupted sensory gating via a similar pathway through serotonin release that resulted in increased dopamine release in the nucleus accumbens. Moreover, the results of the current study support previous findings [41] that haloperidol was able to reverse this disruption. Combined with the current findings, this highlights that dopamine D2 receptor may be crucial to auditory sensory gating. 

SCH23390 pretreatment was effective in blocking the MDMA-induced sensory gating disruption, which suggests that the dopamine D1 receptor is involved in this process. In contrast to all the other cohorts, the SCH23390-pretreated rats had MDMA-induced gating disruptions that were due to an increase in the S2 response as opposed to a decrease in the S1 amplitude. The reason for this difference is unknown, but it may indicate an alternative mechanism. de Bruin et al. [41] suggested that S1 alterations were due to dopaminergic activity, while S2 changes were adrenergic, therefore, there may be other mechanisms involved that were not investigated in the current study. Moreover, SCH23390 pretreatment alone also increases the amplitude of S1 and S2 responses independent of a change in the S2:S1 ratios. A study by McLean et al. [42] found that administration of a dopamine D1 receptor agonist, SKF38393, reversed novel object recognition and reversal learning deficits in rats induced by the glutamate NMDA receptor antagonist, PCP. These deficits were re-induced by administration of SCH23390, however, there was also a trend for SCH23390 to impair cognition when administered alone. This suggests that, like in the current study, blocking dopamine D1 receptors with SCH23390 may influence information processing and cognitive processes.

Dopamine D1 receptors are most abundant in cortical regions. We previously found that systemic administration of a dopamine D1/D2 agonist, apomorphine, caused significant disruptions to PPI but these effects were greater after pretreatment with SCH23390 locally injected into the frontal cortex. These findings suggested that medial prefrontal dopamine D1 receptor activity is critical to PPI regulation [43] and thus may also be crucial to other forms of sensory information processing, which is supported by the results of the current study. Conversely, Gogos et al. [44] found that haloperidol, but not SCH23390, pretreatment could reverse a deficit of PPI induced by 8-OH-DPAT, suggesting that dopamine D2 and not dopamine D1 receptors are involved in sensory gating. However, considering the current results that indicate 5-HT1A, dopamine D2 and dopamine D1 receptors in addition to 5-HT2A receptors are all associated in a common pathway, dopamine D1 receptors may be implicated in this type of sensory gating but not necessarily PPI.

### 4.2. Interactive Effects of D1 and D2 Receptors and of 5-HT2A and 5-HT1A Receptors

In addition to the sequential activation of serotonin and dopamine pathways, activation of all four receptors appeared required to cause the MDMA-induced disruption of auditory sensory gating, and thus, antagonism at any of the four investigated receptors blocked the pathway required to cause a disruption. This implies interactive effects of dopamine D1 and D2 receptors, and of 5-HT2A and 5 HT1A receptors.

Previous research has suggested that 5-HT2A and 5-HT1A receptors have opposing actions [18,39]. In contrast, the current results indicate that the two receptors have analogous actions. The reason for the discrepancies between previous results and the current results is unclear but could be related to the behavioural paradigm studied. The fact that both DOI and 8-OH-DPAT can independently disrupt auditory gating [25] suggests that in this paradigm 5-HT2A and 5-HT1A receptors have similar actions to one another. It has also been shown that there is an interdependence of 5 HT1A and 5-HT2A receptors in several other behavioural paradigms. Already in 1989, Arnt and Hyttel showed that DOI administration facilitated the effect of 8-OH-DPAT on forepaw treading although 8 OH-DPAT inhibited the effect of DOI on head twitches [45,46]. Interestingly, 5-HT2A receptor-mediated head twitches were not only antagonized by 5-HT1A receptor agonists, but also by dopamine D1 receptor and dopamine D2 receptor antagonists [47]. More recently, 5-HT2A and 5-HT1A receptor interaction was shown in hypothalamo-pituitary-adrenal axis activity [48], drug discrimination [49] and frontal cortical pyramidal neurons [50].

Previous research also demonstrated interactive effects of dopamine D1 and dopamine D2 receptors, for example synergistic activation of these receptors is required for a disruption of PPI [51]. These authors found that co-administration of the dopamine D2 agonist, quinpirole, and dopamine D1 receptor agonist, SKF38393, resulted in a disruption of PPI that was not seen when these receptor agonist drugs were administered alone. This result suggests that simultaneous dopamine D2 and dopamine D1 receptor activation may be required to cause a disruption to sensory gating. Similarly, in the current study, combined dopamine D2 and dopamine D1 receptor activation may have resulted in the disruption of paired-click sensory gating induced by MDMA and blocking either of these receptors inhibits the MDMA-induced disruption. 

Overall, it is possible that MDMA administration initiates serotonin release from projections of neurons in the raphe nucleus. This serotonin release activates 5-HT receptors, including 5-HT2A and 5-HT1A receptors in the medial prefrontal cortex and VTA. Subsequently, dopamine levels are also altered which results in activation of dopamine D2 and dopamine D1 receptors, likely in the nucleus accumbens or prefrontal cortex, such that the activation of both dopamine D2 and D1 receptors ultimately causes the sensory gating disruption observed. In order to obtain better spatial information regarding areas of the brain and the associated pathway involved in this MDMA induced auditory sensory gating disruption, future studies should incorporate the use of localised injections, as well as subcortical electrodes. Localised injections into the brain could provide information about where the actions of the drugs originate from and which receptor subtypes are acting to affect auditory gating. The use of subcortical electrodes implanted into specific regions of the brain would elucidate which area of the brain is responsible for the gating responses to narrow down the possible pathways involved. The findings could further clarify convergent and divergent mechanisms of PPI and paired-click sensory gating found by Swerdlow et al. [8], who reported that PPI and sensory gating disruptions induced by apomorphine, PCP and DOI revealed different neurobiological mechanisms of the two sensory gating measurement paradigms. Finally, co-administration of DOI and WAY100635, as well as 8-OH-DPAT and ketanserin could establish whether 5-HT2A and 5-HT1A receptors do, in fact, have analogous, and not opposing actions in sensory gating. 

A limitation of this study is that, while effects on S2:S1 ratio were highly consistent between the different cohorts and drug studies, there was variability in the amplitude of the individual P and N components of the waveforms, resulting in variability of drug effects on S1 vs. S2. It is unclear why there was this inconsistency although previous studies similarly found some variability of effects on waveform components when comparing MDMA with other serotonergic compounds [25] or following changes in experimental protocol [6,8,52]. Future studies should aim to replicate the present results in larger animal cohorts to reduce experimental variability and more clearly associate drug effects to individual waveform components. 

It should furthermore be noted that SCH23390 has some affinity for 5-HT2A receptors [53]. Therefore, the effect of SCH23390 may be partially due to it blocking 5-HT2A receptors rather than dopamine D1 receptors. However, the affinity of SCH23390 for dopamine D1 receptors is 10 to 100-fold greater compared to 5-HT2A receptors [53], thus it is likely that dopamine D1 receptors are involved in the effect of SCH23390 in reversing the MDMA-induced sensory gating disruption. In a study by Schindler et al. [37], DOI- and lysergic acid diethylamide- (LSD) induced head bobs in rabbits were reduced by pretreatment with SCH23390, as well as the 5-HT2A receptor antagonist, ritanserin. This highlights a role for dopamine D1 receptors in this 5-HT2A receptor-mediated hallucinogenic behaviour. The current results suggest that while 5-HT2A receptor-mediated serotonin release may indirectly stimulate dopamine D1 receptor activation to cause an auditory sensory gating disruption, 5-HT1A and dopamine D2 receptors are also involved in the process. 

## 5. Conclusions

The current study has demonstrated that MDMA has profound effects on both serotonin and dopamine systems. Several dopamine and serotonin receptor subtypes are involved in the effects of MDMA that may contribute to the wide array of symptoms, such as both mood and perceptual effects that may underlie some cognitive deficits seen with MDMA use. The results of the current study also provide insight into this sensory gating disruption seen in schizophrenia and mechanisms involved for a greater understanding of deficits in this mental illness. The results of the current study support the involvement of both serotonin and dopamine systems in this information processing deficit. Moreover, they support serotonin and dopamine dysfunction in the pathophysiology of schizophrenia and the use of atypical antipsychotic drugs with both dopaminergic and serotonergic mechanisms of action as treatment for schizophrenia preferential to first-generation antipsychotic medications. 

The current results, moreover, suggest that serotonin and dopamine systems act closely with one another. In fact, 5-HT2A, 5-HT1A, dopamine D2 and dopamine D1 receptors may act synergistically and in series in a common pathway that can cause a deficit to auditory sensory gating in the rat, highlighting the involvement of a complex circuitry. It is hypothesised that dopamine receptors act downstream to serotonin receptors, such that 5 HT2A and 5-HT1A receptors can mediate dopamine release, which acts upon dopamine D2 and dopamine D1 receptor activity.
